# Quercetin promotes the proportion and maturation of NK cells by binding to MYH9 and improves cognitive functions in aged mice

**DOI:** 10.1186/s12979-024-00436-1

**Published:** 2024-05-10

**Authors:** Tingting Su, Haitao Shen, Mengyuan He, Shanshan Yang, Xue Gong, Ce Huang, Liuling Guo, Hao Wang, Shengyu Feng, Taotao Mi, Meili Zhao, Qing Liu, Fengjiao Huo, Jian-Kang Zhu, Jianbo Zhu, Hongbin Li, Hailiang Liu

**Affiliations:** 1 Key Laboratory of Xinjiang Phytomedicine Resource and Utilization of Ministry of Education, College of Life Sciences, Shihezi University, Shihezi, 832003 China; 2 Institute for Regenerative Medicine, State Key Laboratory of Cardiology and Medical Innovation Center, Shanghai East Hospital, School of Medicine, Tongji University, Shanghai, 200123 China; 3 Institute of Advanced Biotechnology and School of Medicine, Southern University of Science and Technology, Shenzhen, 518055 China

**Keywords:** Quercetin (PubChem CID: 5280343), Hematopoietic stem cells, NK cells, MYH9, Cognitive level

## Abstract

**Background:**

Quercetin is a flavonol compound widely distributed in plants that possesses diverse biological properties, including antioxidative, anti-inflammatory, anticancer, neuroprotective and senescent cell-clearing activities. It has been shown to effectively alleviate neurodegenerative diseases and enhance cognitive functions in various models. The immune system has been implicated in the regulation of brain function and cognitive abilities. However, it remains unclear whether quercetin enhances cognitive functions by interacting with the immune system.

**Results:**

In this study, middle-aged female mice were administered quercetin *via* tail vein injection. Quercetin increased the proportion of NK cells, without affecting T or B cells, and improved cognitive performance. Depletion of NK cells significantly reduces cognitive ability in mice. RNA-seq analysis revealed that quercetin modulated the RNA profile of hippocampal tissues in aging animals towards a more youthful state. In vitro, quercetin significantly inhibited the differentiation of Lin^−^CD117^+^ hematopoietic stem cells into NK cells. Furthermore, quercetin promoted the proportion and maturation of NK cells by binding to the MYH9 protein.

**Conclusions:**

In summary, our findings suggest that quercetin promotes the proportion and maturation of NK cells by binding to the MYH9 protein, thereby improving cognitive performance in middle-aged mice.

**Graphical abstract:**

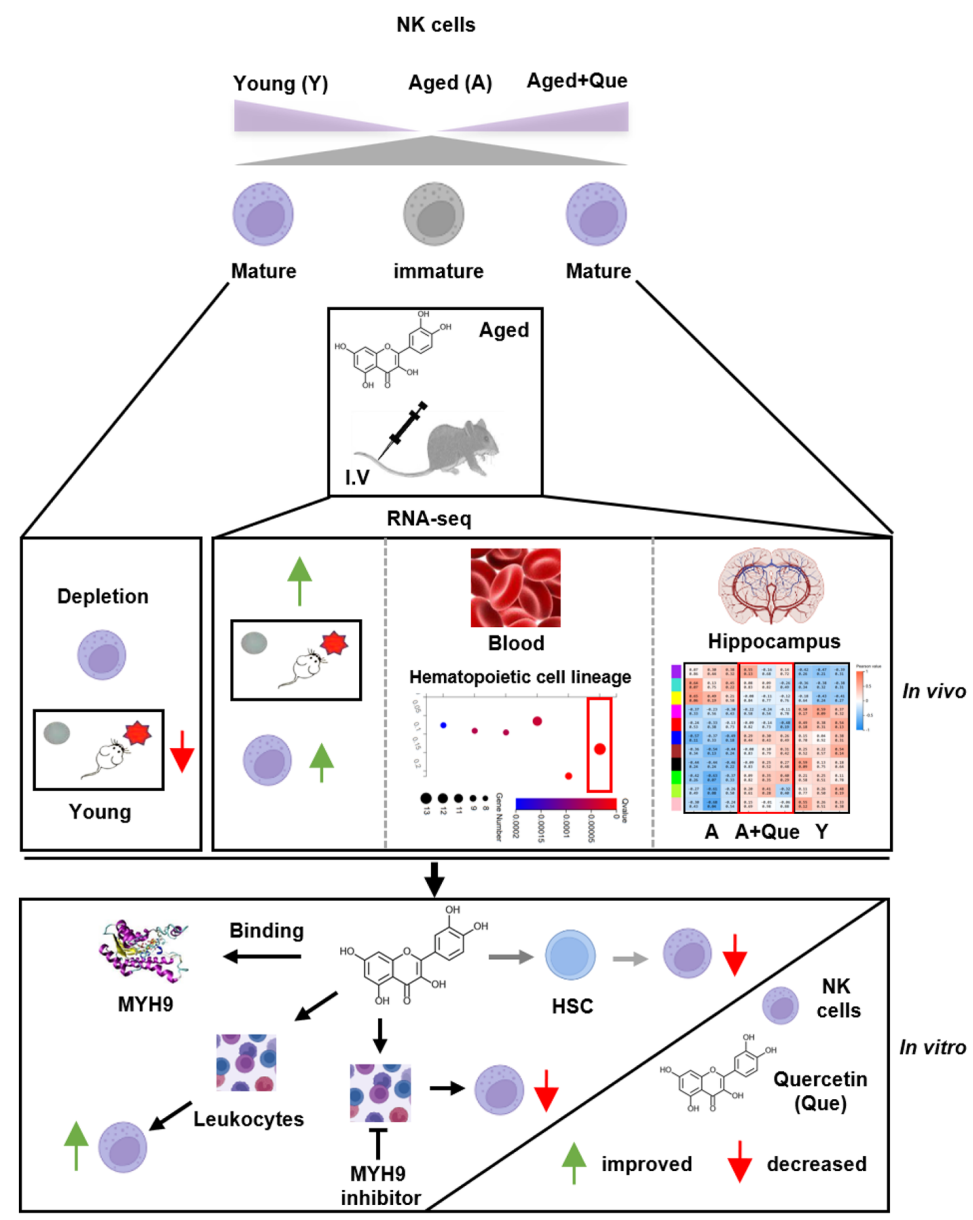

**Supplementary Information:**

The online version contains supplementary material available at 10.1186/s12979-024-00436-1.

## Background

Aging is the consequence of the progressive functional decline of the organism and is a primary risk factor for the development of cancer, cardiovascular disease, memory loss, and various neurodegenerative diseases in the elderly population. Cognitive decline is a key characteristic of aging in humans [1]. Senescence-related neurological disorders in humans, such as Alzheimer’s disease, Huntington’s disease, and Parkinson’s disease, are associated with pathological cognitive decline. Normal aging in humans is also associated with a variety of changes, including cognitive decline, reduced mobility, and an increased prevalence of anxiety and depression.

Immunosenescence is the aging-related impairment of the immune system, and is associated with a reduced ability to respond to infections, deterioration of long-term immune memory, and dysregulation of immune surveillance [2]. Studies show that the immune system plays a critical role in the aging process and is closely linked to age-related diseases. For example, in a murine model of immune system senescence, the mice exhibited generalized senescence, and transplantation of their immune cells into wild-type animals accelerated senescence, shortening the lifespan of the recipient mice [3]. Moreover, improving immune function and redox status in mice with early senescence increases their life span [4]. Furthermore, a growing body of evidence shows that the immune system can regulate brain functions and ameliorate neurodegenerative disease symptoms and cognitive impairment [5, 6]. Indeed, immunodeficient mice exhibit marked deficits in learning and cognitive functions [7]. IL-4 (from T-cells) enhances cognitive ability in immunocompromised mice by modulating myeloid lineage (M2) cells [8]. Additionally, NK cells effectively remove α-synuclein aggregates and protect against neurodegenerative disorders, such as Parkinson’s disease and dementia with Lewy bodies, which are associated with pathological synuclein deposits [9].

In recent years, small molecule compounds have become a hotspot in several research fields for their numerous advantages, including natural origin, multi-target and multiple pathways of action, and few side effects. Quercetin is a flavonol compound with multiple biological activities that is widely distributed in the plant kingdom and present in a variety of foods, such as vegetables, fruits and tea [10]. Quercetin has a variety of biological effects in vivo and in vitro, including antioxidant [11], anti-inflammatory [12], anticancer [13–15], neuroprotective [16, 17] and anti-aging actions [18, 19]. Quercetin exerts its neuroprotective effects by targeting a variety of signaling pathways, including PI3K/AKT [20], JNK [21], Nrf2-ARE [22, 23], PNO2 [24] and MAPK [25]. Moreover, quercetin ameliorates age-related neurological diseases, including Alzheimer’s disease, Parkinson’s disease [26], and chronic cerebral ischemia [27], by activating the AMPK signaling pathway, reducing mitochondrial dysfunction [28] and enhancing oxidative defense mechanisms [29], thereby improving learning and memory functions. Additionally, quercetin has immunomodulatory effects. Quercetin inhibits lipopolysaccharide (LPS)-induced tumor necrosis factor (TNF-α) production in macrophages [30]. It also inhibits LPS-induced increases in the mRNA levels of TNF-α and interleukin-1α in glial cells [31], and it enhances the lytic activity of NK cells [32]. However, whether quercetin affects cognitive functions by modulating the immune system remains unclear. Therefore, in the present study, we investigate changes in cognitive abilities in aging mice treated with quercetin, and we analyze immune cell alterations in peripheral tissue, specifically, the spleen. We anticipate that the findings will provide a foundation for future studies on quercetin as a therapeutic agent for age-related cognitive and memory disorders.

## Results

### Quercetin improves short-term memory ability and increases the proportion of NK cells in middle-aged mice

**Fig. 1:**
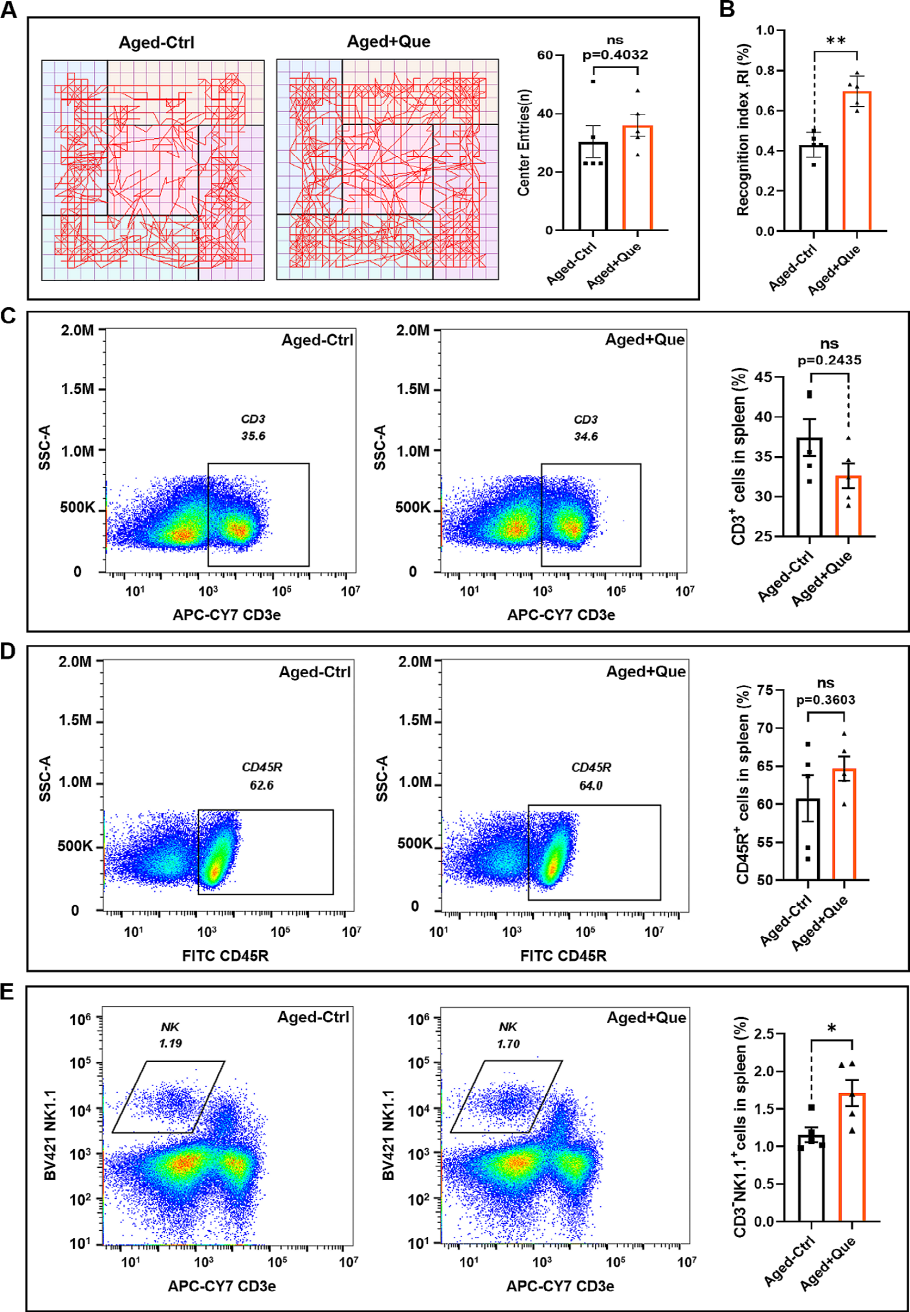
Quercetin improves cognitive ability and increases the number of NK cells in middle-aged mice. (**A**) Representative path plots and the number of crossings of the central region in the open field test for quercetin-treated (Que) and control mice. (**B**) Recognition indexes of the Que and control groups in the novel object recognition experiment. (**C**) Representative flow cytometry plots and bar graphs of T cells in the spleens of Que and control groups of mice. (**D**) Representative flow cytometry plots and histograms of B cells in the spleens of Que and control groups of mice. (**E**) Representative flow cytometry plots and histograms of NK cells in the spleens of the Que and control groups. (*n* = 5 for each group, ns *p* > 0.05, *p < 0.05, **p < 0.01)

We investigated anxiety/depression-like behaviors and the learning and memory abilities of middle-aged mice (10 months) treated with quercetin via tail vein injection (1 mg/kg every 2 days for 30 days) using the novel object recognition and open field tests. There was no significant difference in the number of crossings of the center area between the quercetin-treated and control groups in the open field test (Fig. 1A). Quercetin did not affect the anxiety levels of middle-aged mice, but in the novel object recognition test, the recognition index was significantly higher in the quercetin-treated group than in the control group (Fig. 1B). This result suggests that quercetin significantly improved the short-term memory ability of middle-aged mice. As mice age, the quantity and functions of immune cells undergo changes. Our previous studies demonstrated an association between immune cells and cognitive function [33, 34]. Therefore, we examined the immune cell population in the spleen after quercetin treatment. Compared with the control group, the proportions of T and B cells in the quercetin-treated mice were not significantly different (Fig. 1C, D). However, there was a significant increase in the proportion of NK cells (Fig. 1E, F). These findings suggest that quercetin treatment had no impact on the splenic T and B cell populations, but increased the proportion of NK cells. Therefore, the influence of quercetin on short-term memory in middle-aged mice may be related to its effect on NK cells.

### Quercetin promotes the maturation of NK cells

**Fig. 2:**
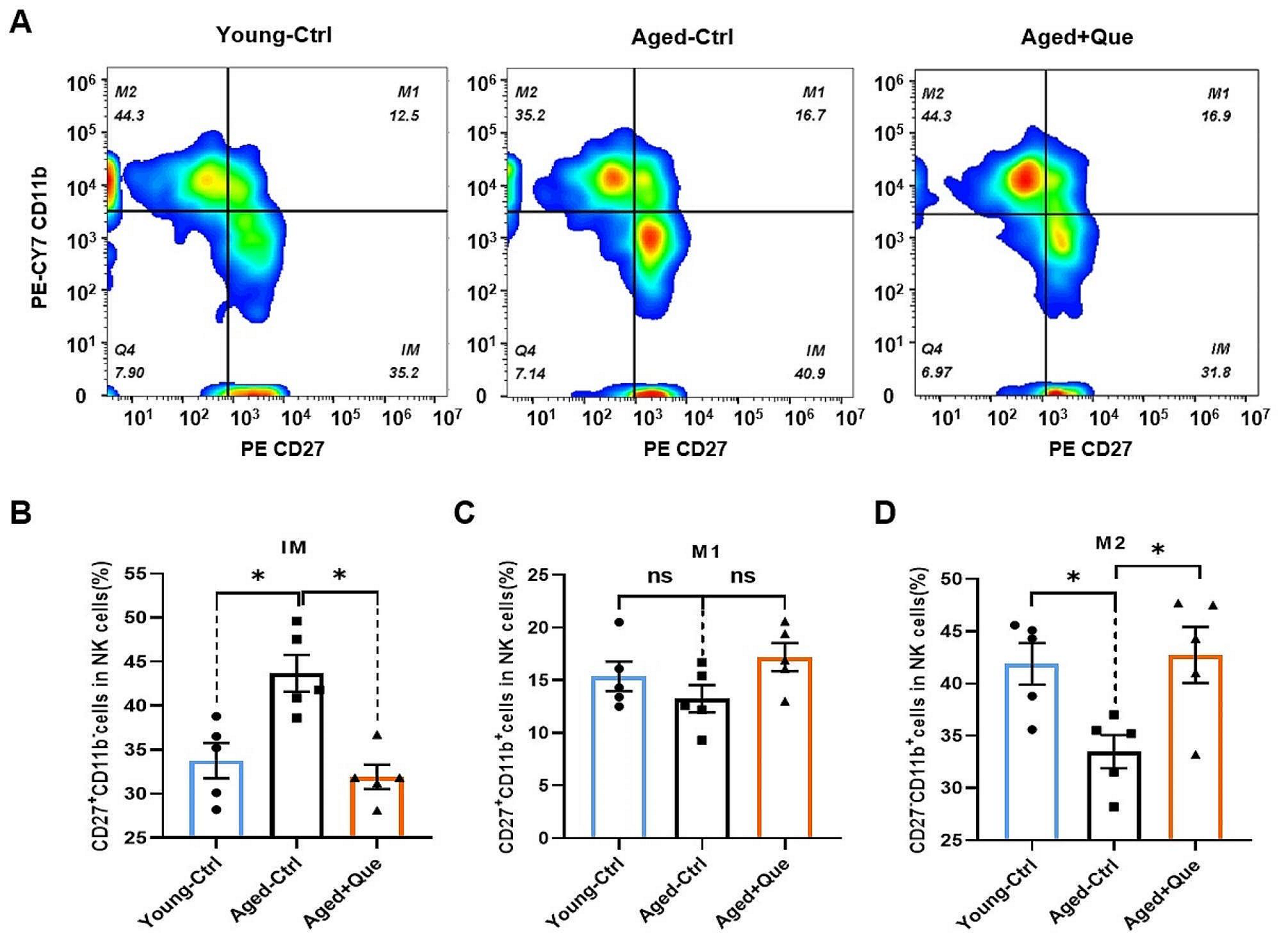
Quercetin promotes NK cell maturation in vivo. (**A**) Representative flow cytometry profiles of NK cell subsets in the spleens of young control, middle-aged control (Ctrl) and quercetin-treated (Que) mice. (**B**) Proportions of CD27^+^CD11b^−^ NK cells (immature, IM). (**C**) Proportions of CD27^+^CD11b^+^ NK cells (intermediate mature, M1). (**D**) Proportions of CD27^−^CD11b^+^ NK cells (mature, M2). (*n* = 5 for each group, ns *p* > 0.05, *p < 0.05)

NK cells are components of the innate immune system, but they also participate in the adaptive immune system and in the immune memory response [35]. Mouse NK cells were identified by CD3^−^NK1.1^+^ labeling, and mature functional NK cells were divided into three different subsets according to their expression of CD27 and CD11b, as follows: CD27^+^CD11b^−^ (immature), CD27^+^CD11b^+^ (intermediate mature) and CD27^−^CD11b^+^ (mature) [36]. CD27^+^CD11b^−^ cells represent immature NK cells with potent proliferative ability. CD27^+^CD11b^+^ cells are intermediate mature NK cells with the highest cytotoxicity. CD27^−^CD11b^+^ cells are terminal mature NK cells with a strong ability to secrete cytokines. To further investigate the effects of quercetin on NK cells, we conducted a quantitative analysis of NK cell subsets in the spleen following quercetin treatment. Flow cytometry showed that, compared with young mice, the proportion of early CD27^+^CD11b^−^ NK cells were increased significantly in middle-aged mice (Fig. 2A, B). There was no statistically significant difference in the proportion of intermediate phase CD27^+^CD11b^+^ NK cells (Fig. 2C), but there was a significant decrease in the proportion of terminally mature CD27^−^CD11b^+^ NK cells (Fig. 2D). Following quercetin treatment in middle-aged mice, the proportion of early CD27^+^CD11b^−^ NK cells decreased, while the proportion of terminal mature CD27^−^CD11b^+^ NK cells increased. These results indicate that quercetin promotes the maturation of NK cells.

### Quercetin enhances the proportion of NK cells and facilitates NK cell maturation in vitro

**Fig. 3:**
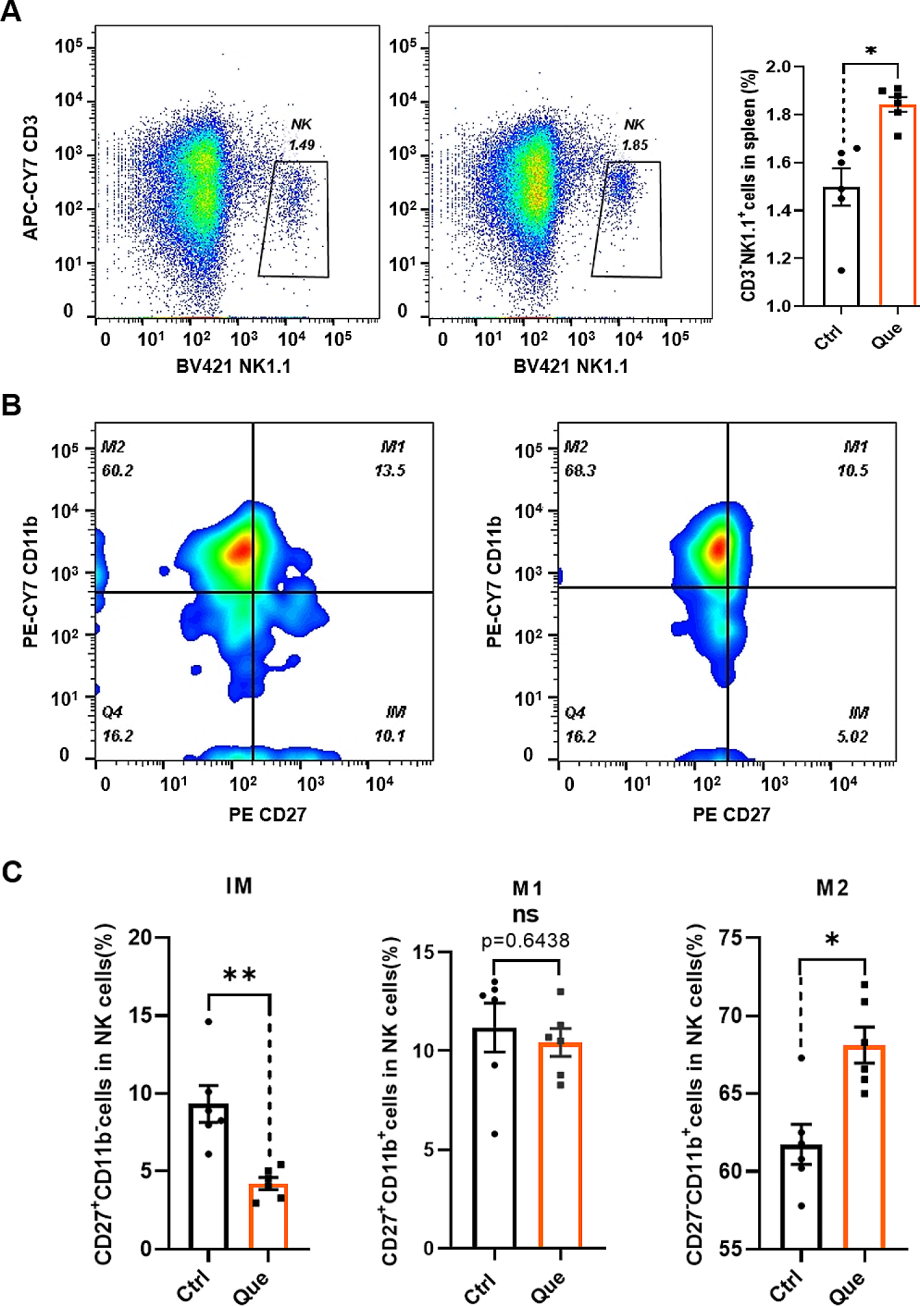
Quercetin promotes the proportion and maturation of splenic NK cellsin vitro. Cells were treated with 25 µM quercetin (Que) or DMSO (Control). (**A**) Representative flow cytometry profiles and bar graphs of splenic NK cells. (**B**) Representative flow cytometry profiles of NK cell subpopulations (immature cells, IM; intermediate mature cells, M1; mature cells, M2). (**C**) The proportions of CD27^+^CD11b^−^ (IM), CD27^+^CD11b^+^ (M1) and CD27^−^CD11b^+^ (M2) NK cell subpopulations. Data represent mean ± SEM. *P < 0.05. **p < 0.01. ns, not significant, vs. Ctrl

Aging leads to a decline in the self-renewal ability of hematopoietic stem cells (HSCs) in the body, resulting in an increased number of HSCs differentiating into myeloid lineage cells, a decreased number of HSCs differentiating into gonadal lineage cells, and the reduced production of immune cells [37]. The NK cells in peripheral tissues observed were derived from HSCs. The above findings demonstrate that quercetin increases the proportion of NK cells and promotes NK cells differentiation in vivo. We therefore investigated whether quercetin predominantly impacts the proportion or maturation of NK cells. The effect of quercetin on NK cells was investigated by treating spleen mononuclear cells in vitro using four different concentrations of the compound (3.125, 6.25, 12.5 and 25 µM). These concentrations had no significant effect on T or B cells. However, 12.5 and 25 µM quercetin markedly increased number of NK cells (Fig. S1). Thus, a concentration of 25 µM was used for subsequent experiments. Quercetin at 25 µM not only promoted NK cell number (Fig. 3A), but also reduced the number of early CD27^+^CD11b^−^ cells and increased the number of terminally mature CD27^−^CD11b^+^ cells (Fig. 3B, C). These findings are consistent with the in vivo results obtained from quercetin-treated mice, suggesting that quercetin increases the percentage of NK cells and promotes NK cells differentiation in the spleen.

### Quercetin inhibits the differentiation of HSC-derived NK cells

**Fig. 4:**
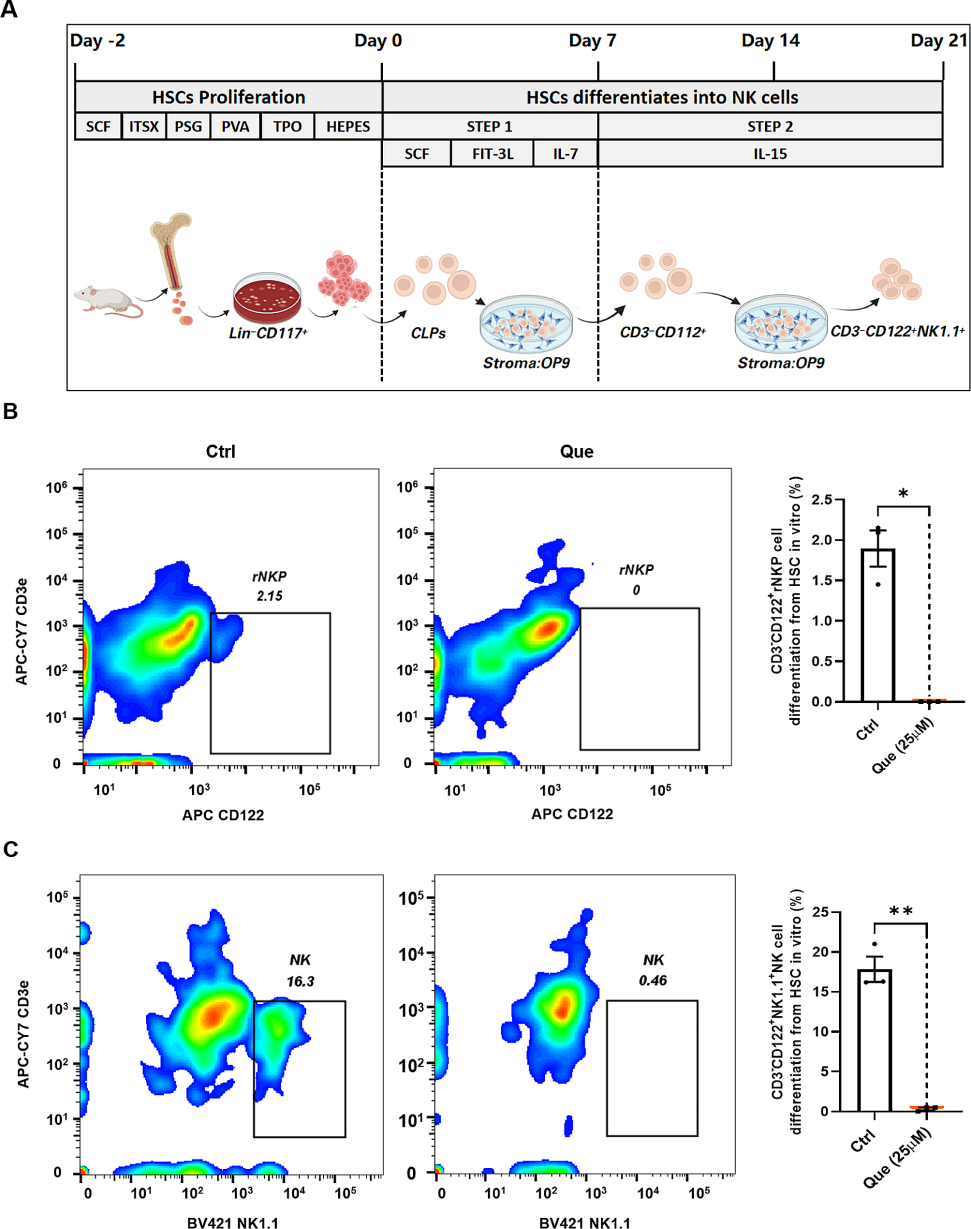
Quercetin inhibits the differentiation of HSCs to NK cells. (**A**) Culture protocol for the differentiation of Lin^−^CD117^+^ hematopoietic stem cells into NK cells. (**B**) Representative flow cytometry profiles and bar graphs for the differentiation of 25 µM quercetin-treated (Que) and DMSO-treated (Control) Lin^−^CD117^+^ hematopoietic stem cells into rNKP cells. (**C**) Representative flow cytometry profiles and bar graphs for the differentiation of Que and Control Lin^−^CD117^+^ hematopoietic stem cells into NK cells. Data represent mean ± SEM. *P < 0.05. **p < 0.01

To examine the effect of quercetin on the differentiation of HSC-derived NK cells, we conducted in vitro differentiation experiments. To ensure that the concentration of quercetin was non-cytotoxic and did not induce cellular damage, three concentrations of quercetin (6.25, 12.5 and 25 µM) were selected for treating HSCs during the proliferative period, and their viability was assessed using the CCK-8 assay. None of the three concentrations of quercetin exhibited cytotoxicity towards HSCs. A concentration-dependent increase in HSC proliferation was observed (Fig. S2). Therefore, 25 µM quercetin was chosen for the following experiments. HSCs obtained from bone marrow were sorted to obtain Lin^−^CD117^+^ cells, which were then co-cultured with OP9 cells for 21 days. The culture medium was supplemented with appropriate differentiation factors to establish an in vitro culture model of HSC-to-NK cell differentiation. Following 7 days of culture, HSCs differentiated into common lymphoid progenitor (CLP) cells, which subsequently matured into CD122^+^ rNKP cells after an additional 7 days of culture. The rNKP cells then proceeded to differentiate into NK1.1^+^ NK cells (Fig. 4A). Flow cytometry analysis was used to examine CD122^+^ rNKP cells on the 14th day of differentiation culture, as well as NK1.1^+^ NK cells on the 18th day. After 14 days of differentiation, the Control group contained approximately 2% CD3^−^CD122^+^ rNKP cells, whereas the quercetin group did not contain any CD122^+^ cells (Fig. 4B). On the 21st day of differentiation, the CK group contained approximately 16% NK1.1^+^ cells, indicating successful differentiation of HSCs into NK cells. In contrast, the quercetin group did not express CD122 or NK1.1, suggesting the absence of NK cell differentiation from HSCs (Fig. 4C). Together, these in vitro findings suggest that quercetin does not promote the differentiation of HSCs into NK cells, but instead significantly impedes the differentiation of NK cells derived from HSCs.

### Quercetin targets the MYH9 protein

**Fig. 5:**
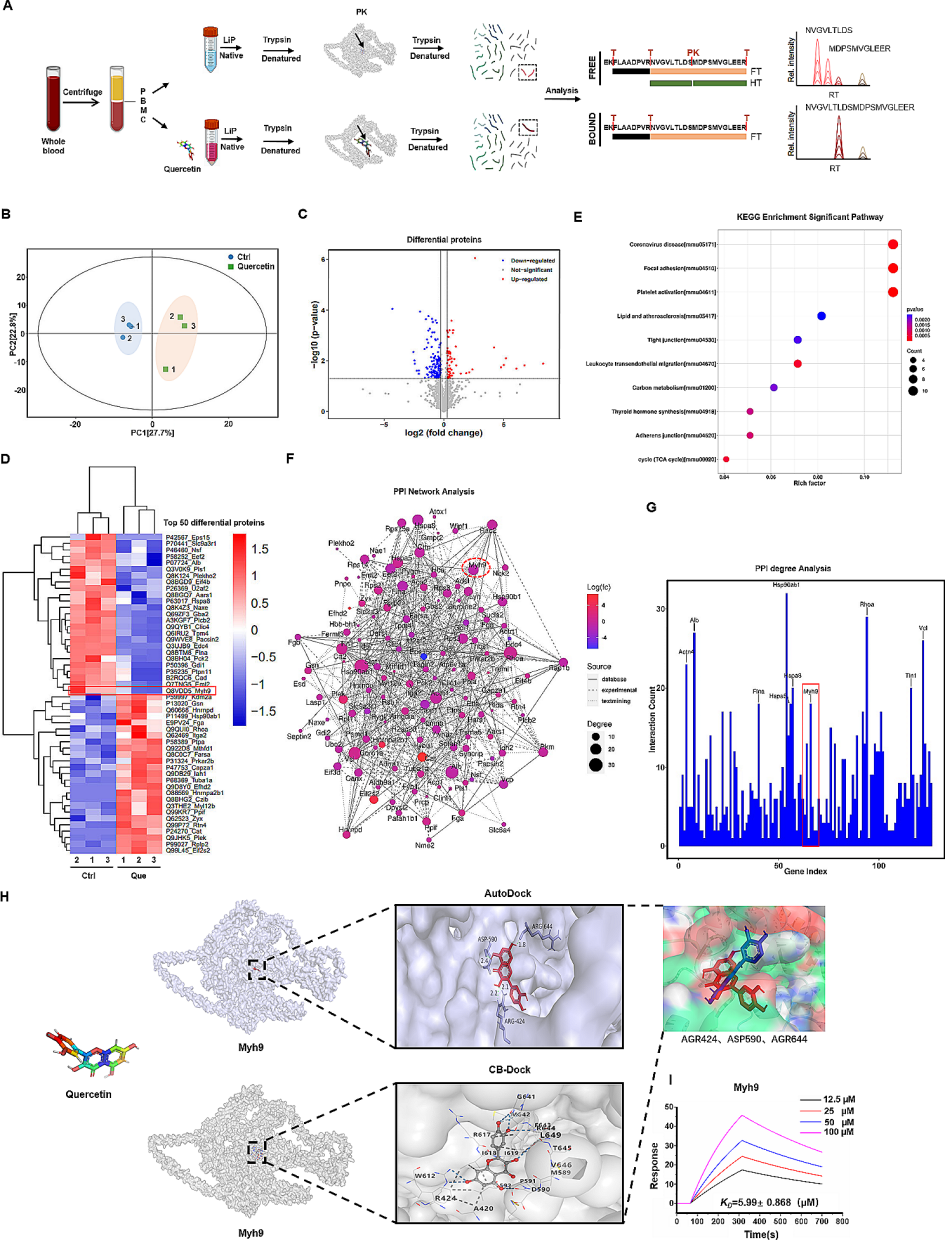
Quercetin binds to MYH9 protein. (**A**) Label-free quantitative (LFQ) proteomics study. Schematic of the experimental design of nanoLC-mass spectrometry analysis for investigating the interaction between quercetin (Que) and PBMC proteins. (**B**) Principal component analysis (PCA) plot of control and quercetin-treated samples. (**C**) Volcano plot of the differential expression of peptides between the quercetin-treated and control groups. (**D**) Heatmap representation of the differentially-expressed peptides. (**E**) KEGG analysis of the differentially-expressed peptides. (**F**) Protein–protein interaction (PPI) network analysis of the differentially-expressed proteins. (**G**) Histogram displaying the connectivity of interacting proteins in the differentially-expressed protein set. The y-axis shows the number of proteins involved in the protein–protein interactions. (**H**) Visualization of the predicted binding sites for quercetin and MYH9 protein using AutoDock and CB-Duck. (**I**) Binding affinity of SPR COOH and MYH9-quercetin interactions. MYH9 was immobilized on COOH chips with quercetin concentrations (from top to bottom) of 100, 50, 25 and 12.5 µM

To further clarify the effects of quercetin on immune cells and uncover its target proteins and associated signaling pathways, we isolated PBMCs from C57BL/6 mice. Total cellular protein from PBMCs was extracted, and the interaction between quercetin and the extracted total protein was evaluated using Metpro protein–ligand interaction detection technology. Following incubation with quercetin, total cellular protein was enzymatically digested with trypsin, and the resulting peptides were eluted and analyzed using nanoLC-MS/MS, which successfully identified a total of 667 proteins and 1,596 peptides (Fig. 5A). To assess similarities and differences between the groups, we performed principal component analysis (Fig. 5B). In addition, differential screening of the detected peptide fragments was performed, resulting in the identification of 189 differentially-expressed peptides between the control and quercetin-treated groups (Fig. 5C and Fig S3). Subsequently, the 50 peptides displaying the most significant differential expression levels were subjected to hierarchical cluster analysis and visualized by heat map (Fig. 5D). This revealed a significant enrichment of multiple proteins associated with the cellular cytoskeleton, including the non-myosin IIA-MYH9 protein, caused by quercetin treatment. Furthermore, these differential peptides were labeled with subordinate proteins, and KEGG analysis was performed to identify significantly-enriched metabolic pathways. The analysis revealed a strong correlation between quercetin and pathways linked to coronavirus diseases, focal adhesions, platelet activation, lipids, and atherosclerosis (Fig. 5E). We constructed an interaction network diagram of the differentially-expressed proteins (Fig. 5F) and analyzed the connectivity between these interacting proteins (Fig. 5G), which revealed an association between quercetin and the MYH9 protein. The MYH9 protein, a fundamental constituent of the cellular skeleton, plays a critical role in preserving and facilitating the functionality of HSCs [38]. Additionally, it exerts influence over immune cells and regulates the cytotoxicity of NK cells [39]. Thus, the MYH9 protein is likely to be a primary target of quercetin. Therefore, we simulated their binding using CB-Duck and AutoDuck molecular docking software. In order to obtain more accurate docking results and prevent blind searching for the optimal ligand conformation throughout the entire protein structure, we initially employed the online molecular docking tool CB-DOCK to predict potential binding sites for quercetin and the protein. Subsequently, AutoDock simulations were performed based on the cavity dimensions of the predicted binding sites. After docking, we chose the docking results with the lowest binding energy and hydrogen bond generation in AutoDock, as well as a shared binding site in CB-DOCK. The simulation results showed that quercetin was located in the same cavity of the MYH9 protein, using both docking tools, with three common binding sites—arginine 424, aspartic acid 590 and arginine 644 (Fig. 5H).

Surface plasmon resonance (SPR) analysis was employed to verify the binding capability of the MYH9 protein to quercetin. Based on the analysis of the binding constant (Ka), dissociation constant (Kd), affinity constant (KD), response curve, and affinity ranking of quercetin for MYH9 protein at varying concentrations, a specific 1:1 binding interaction between quercetin and MYH9 protein was identified. Moreover, the MYH9 response to quercetin displayed a trend towards concentration-dependency (Fig. 5I). These results demonstrate specific binding of quercetin to MYH9. Therefore, quercetin may have potential as a modulator of NK cell function through its specific targeting of MYH9.

### MYH9 protein promotes the proportion and maturation of NK cells

**Fig. 6:**
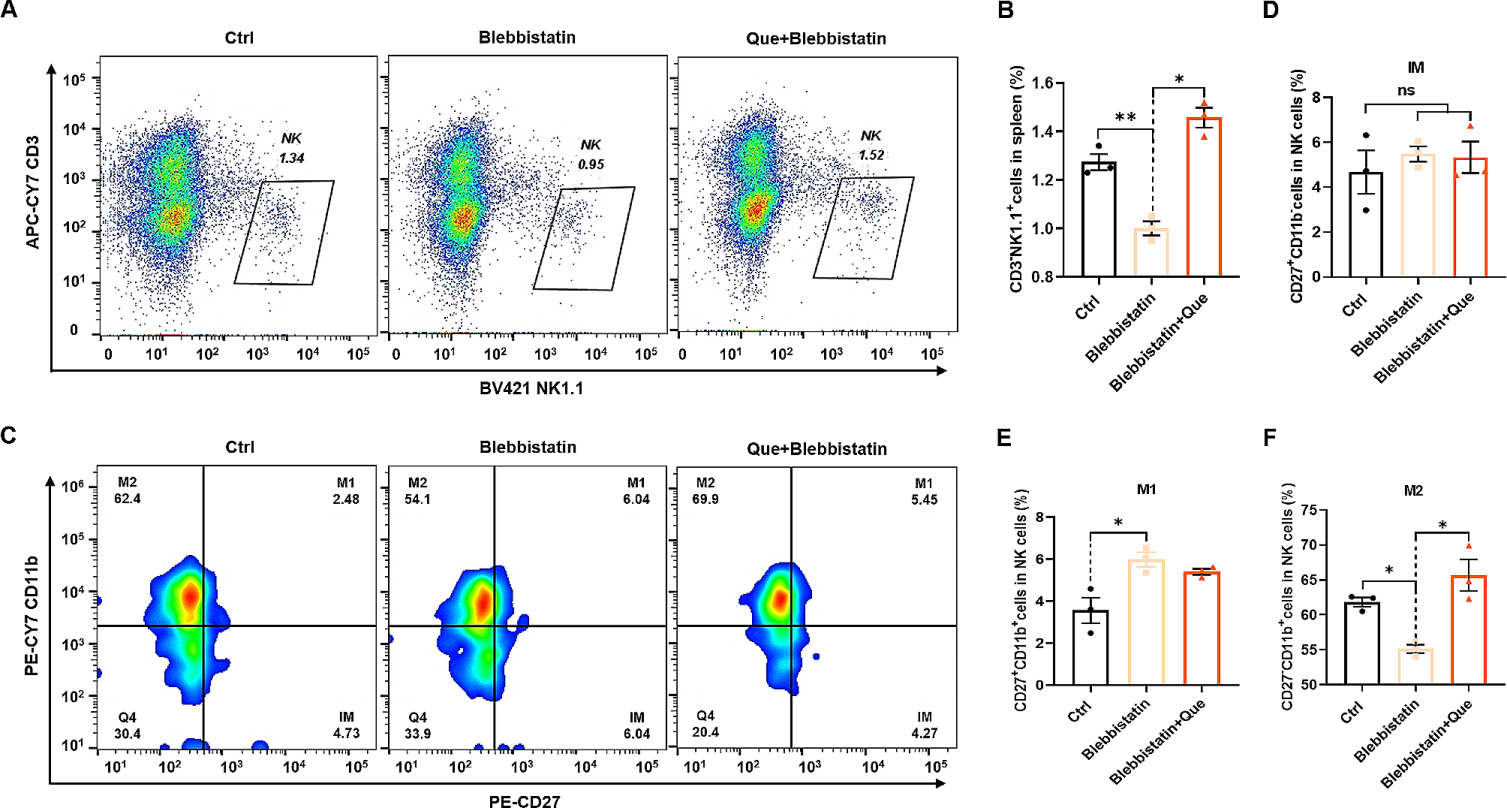
MYH9 protein promotes NK cell proportion and maturation. Representative flow cytometry profiles (**A**) and proportions (**B**) of NK cells among control (Ctrl), blebbistatin-treated (Blebbistatin), and quercetin (Que; 25 µM) + blebbistatin-treated spleen mononuclear cells. **C**, Representative flow cytometry profiles (**C**) and proportions of NK cell subpopulations CD27^+^CD11b^−^ (**D**), CD27^+^CD11b^+^ (**E**) and CD27^−^CD11b^+^ (**F**) among ctrl, blebbistatin, and blebbistatin + Que- treated spleen mononuclear cells. Data represent mean ± SEM. *P < 0.05. **p < 0.01. ns, not significant, vs. Ctrl

To investigate the role of MYH9 in NK cell proportion and maturation, mouse spleen mononuclear cells were treated with the MYH9 inhibitor blebbistatin (10 µM). Flow cytometric analysis was performed to examine the abundance of NK cells, which revealed a significant inhibition of NK cell proportion by blebbistatin compared with the control group (Fig. 6A). Notably, this inhibitory effect was significantly mitigated by treatment with quercetin (25 µM) (Fig. 6B). We also analyzed the NK cell subpopulations (Fig. 6C), which showed that there was no statistically significant difference in the number of early CD27^−^CD11b^+^ NK cells between the control and blebbistatin-treated groups (Fig. 6D), whereas the number of mid-phase CD27^+^CD11b^+^ NK cells was increased (Fig. 6E) and the number of end-phase CD27^−^CD11b^+^ NK cells was decreased (Fig. 6F). Quercetin treatment significantly increased the number of terminally differentiated NK cells (Fig. 6F). These results show that downregulation of MYH9 in splenocytes inhibits the proportion and maturation of NK cells. This inhibitory effect is relieved by quercetin. MYH9 promotes the proportion and maturation of NK cells, and quercetin exerts its regulatory effects on NK cells by binding to MYH9.

### Depletion of NK cells significantly reduces short-term memory ability in mice

**Fig. 7:**
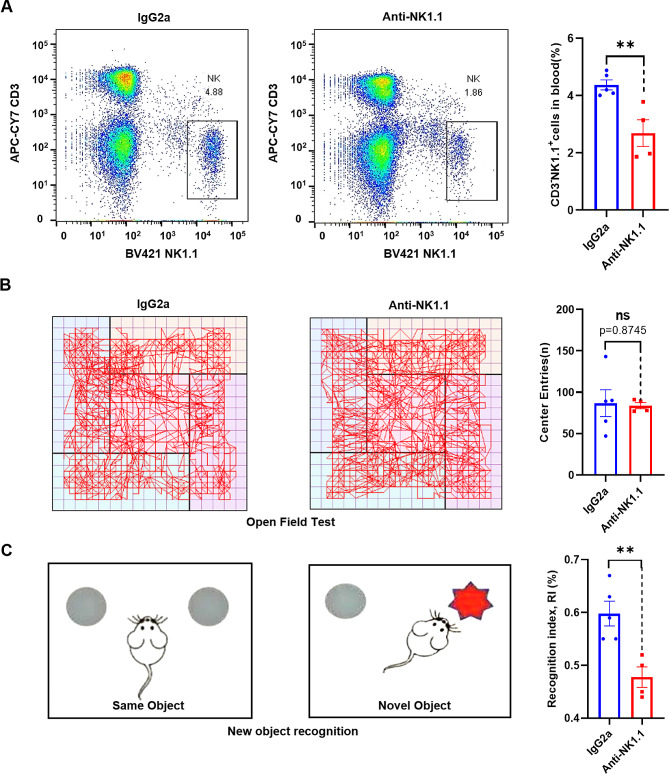
Depletion of NK cells reduces short-term memory ability in mice. (**A**) Representative flow cytometry profiles and bar graphs of NK cells in the spleens of IgG2a and anti-NK1.1 antibody-treated 8-week-old C57BL/6 mice. (**B**) Representative pathway maps and number of crossings of the central area in the open-field test in IgG2a and anti-NK1.1 antibody-treated 8-week-old C57BL/6 mice. (**C**) Schematic of the novel object recognition (NOR) experiment. The cognitive index from the NOR test in IgG2a and anti-NK1.1 antibody-treated 8-week-old C57BL/6 mice. Data represent mean ± SEM. **p < 0.01. ns, not significant, vs. IgG2a

Numerous studies show an involvement of the immune system in the regulation of brain function, with peripheral immune cells capable of impacting cognition by entering the [7]. NK cells have been detected in the brain parenchyma, and they have been demonstrated to participate in neural repair after damage to the CNS, improve brain damage in Parkinson’s disease, and play a neuroprotective role [40]. To further assess the role of NK cells in cognitive function in mice, we depleted NK cells by intraperitoneal injection of anti-NK1.1 antibody [41, 42] and then evaluated cognitive performance. To assess the efficacy of NK cell depletion, we employed flow cytometry analysis to measure the percentage of NK cells in blood after the novel object recognition and open field tests were conducted. The analysis revealed a reduction in the proportion of NK (CD3^−^NK1.1^+^) cells in the group treated with anti-NK1.1 antibody, compared with the control group injected with IgG2a (Fig. 7A). This demonstrates that the mice were lacking NK cells when they performed the novel object recognition and open field tests. During the open field test, no statistically significant difference was observed in the number of center zone crossings between the NK cell depletion group and the isotype control group (Fig. 7B), indicating that NK cell depletion had no impact on anxiety or depressive behaviors in mice. In contrast, the novel object recognition test showed that the cognitive index in the anti-NK1.1 antibody group was decreased compared with the isotype control group (Fig. 7C), suggesting that short-term memory functions were impaired. These findings collectively demonstrate an influence of NK cells on the cognitive abilities of mice.

### Quercetin improves gene expression patterns in the hippocampus of middle-aged mice

**Fig. 8:**
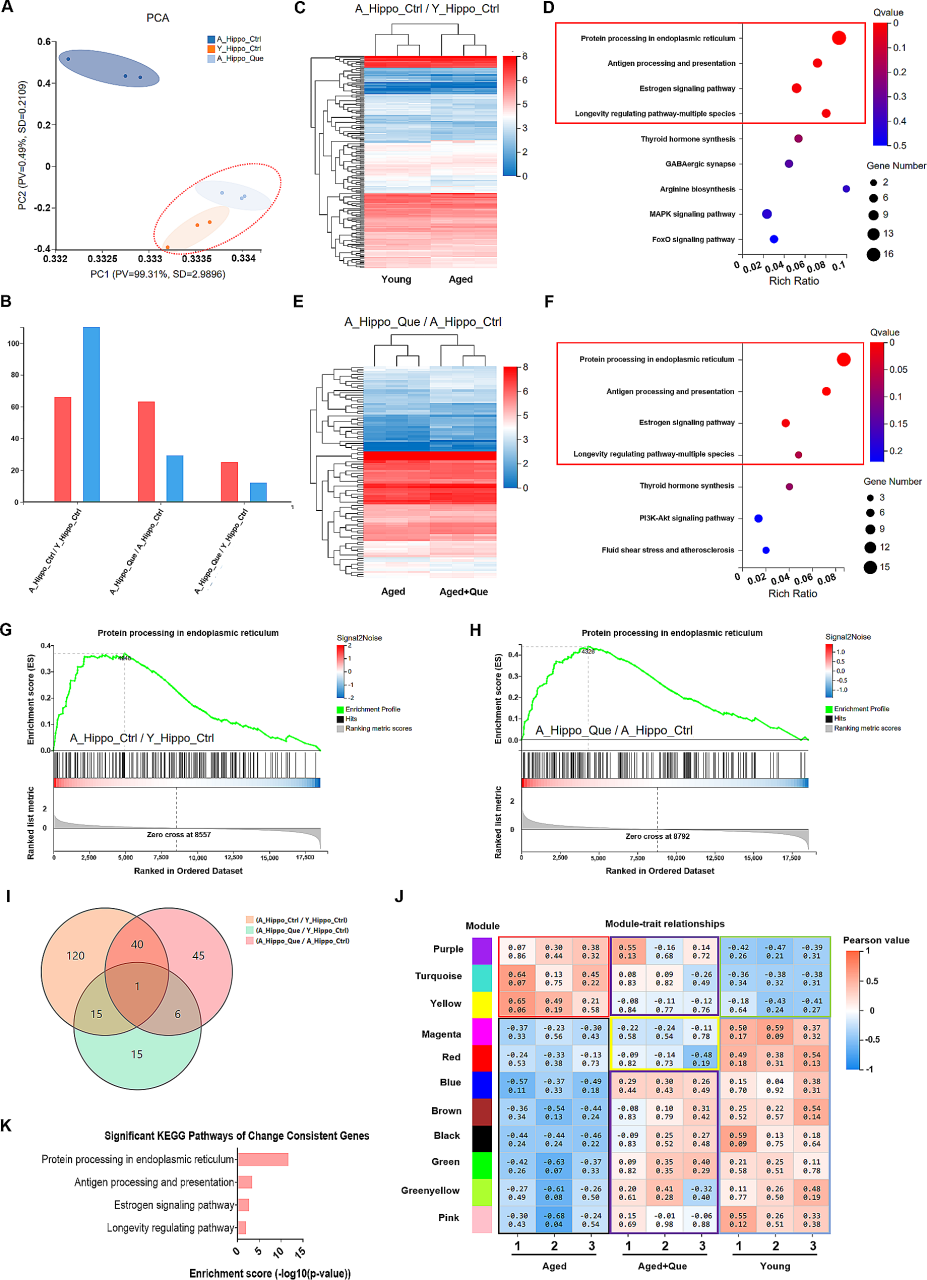
Hippocampal RNA sequencing of young mice, middle-aged mice and quercetin (Que)-treated middle-aged mice. (**A**) Principal Component Analysis (PCA) plots of the A-Hippo-Ctrl, Y-Hippo-Ctrl and A-Hippo-Que samples. (**B**) Number of differentially-expressed genes between each pair of the A-Hippo-Ctrl, Y-Hippo-Ctrl and A-Hippo-Que samples. (**C**) Heatmap of differentially-expressed genes in the A-Hippo-Ctrl and Y-Hippo-Ctrl samples. (**D**) KEGG analysis of the differential genes in A-Hippo-Ctrl and Y-Hippo-Ctrl samples. (**E**) Heatmap of the differential genes in A-Hippo-Ctrl and A-Hippo-Que samples. (**F**) KEGG analysis of the differential genes in the A-Hippo-Ctrl and A-Hippo-Que samples. (**G**) GSEA plot showing enrichment of upregulated genes (red) and repressed genes (blue) at baseline for genes involved in protein processing in the endoplasmic reticulum in A-Hippo-Ctrl compared with Y-Hippo-Ctrl. (**H**) GSEA plot showing the enrichment of genes involved in protein processing in the endoplasmic reticulum in A-Hippo-Que compared with A-Hippo-Ctrl for upregulated genes (red) and repressed genes (blue) at baseline. (**I**) Pie chart showing the number of differentially-expressed genes between each pair of the A-Hippo-Ctrl, Y-Hippo-Ctrl and A-Hippo-Que samples. (**J**) Plot of weighted gene co-expression network analysis (WGCNA) in the samples from the Aged, Aged + Que and Young groups. (**K**) KEGG analysis of genes with altered expression patterns in the hippocampus of quercetin-treated mice

The hippocampus is a crucial region of the brain involved in learning and memory [43]. NK cells have been detected in the dentate gyrus region of the hippocampus [44]. To further investigate the effects of quercetin treatment on the brains of middle-aged mice, we performed RNA-seq analysis of the hippocampus from 8-week-old young mice, 10-month-old middle-aged mice and middle-aged mice given quercetin treatment. The analysis revealed high similarity between the hippocampal samples from the young group and the quercetin-treated middle-aged group by principal component analysis. Moreover, the hippocampal samples from the middle-aged control group differed significantly from the other two groups (Fig. 8A). Next, we conducted pairwise comparison analysis of the three groups. The differentially-expressed genes between each pair of groups were analyzed by hierarchical clustering and visualized by heat map (Fig. 8B). This revealed a substantial number of differentially expressed genes between the young and middle-aged control mice (Fig. 8C). Next, we performed KEGG pathway enrichment analysis on these differentially-expressed genes and discovered that the majority of them were concentrated in pathways related to protein processing in the endoplasmic reticulum, antigen processing and presentation, estrogen signaling pathways, and the multi-species-longevity regulatory pathway (Fig. 8D). There were more differentially-expressed genes between the quercetin-treated middle-aged mice and middle-aged control mice (Fig. 8E), while there were fewer differentially-expressed genes between young mice and quercetin-treated middle-aged mice. Interestingly, we found that the metabolic pathways significantly enriched in the middle-aged control group compared with the quercetin-treated middle-aged group were the same as those enriched in the middle-aged group compared with the young group (Fig. 8F). We also performed gene set enrichment analysis (GSEA) on genes involved in protein processing in the endoplasmic reticulum in the three groups of hippocampal samples. We found a higher expression of genes related to protein processing in the endoplasmic reticulum in both young mice (Fig. 8G) and quercetin-treated middle-aged mice (Fig. 8H), while these genes were expressed at lower levels in the middle-aged control group mice. Importantly, the expression pattern of this gene set was similar between the young group and the quercetin-treated group. In addition, we quantified the overlap of the differential genes between each group pair. There were more common differential genes between the middle-aged control group and the young group, as well as between the middle-aged control group and the quercetin-treated group, but fewer common differential genes between the young group and the quercetin-treated group (Fig. 8I). Weighted gene co-expression network analysis (WGCNA) demonstrated that the middle-aged group showed positive correlations with three gene modules, namely purple, turquoise and yellow, while the young group showed negative correlations. In contrast, the expression patterns of the middle-aged group treated with quercetin exhibited changes within these three gene modules, resulting in reduced correlation (Fig. 8J). Additionally, the blue, brown, black, green, yellow-green and pink gene modules, which demonstrated negative correlations in the middle-aged group, showed decreased correlation after quercetin treatment, with positive correlations similar to those in the young group (Fig. 8J). Finally, by analyzing the metabolic pathways enriched in the gene modules showing altered expression patterns after quercetin treatment, we identified significant overlap of the enriched pathways between the middle-aged control group and the young group, as well as between the middle-aged control group and the middle-aged quercetin-treated group. These findings suggest that there are significant differences in hippocampal gene expression patterns between middle-aged and young mice. Furthermore, quercetin alters the gene expression profiles in middle-aged mice to ones similar to those in young mice.

## Discussion

With the aging of the population, aging-related neurodegenerative diseases, such as Alzheimer’s disease and Parkinson’s disease, have become an increasing burden on patients and society. Quercetin, a small-molecule flavonoid compound, has been shown to improve neurodegenerative diseases through multiple mechanisms of action [45–47] and can be used in combination with dasatinib to selectively remove senescent cells in vivo, reduce microglial activation, and decrease the senescence-associated secretory phenotype to improve cognitive function in mice [48].

Numerous studies show that various immune cell subpopulations in the adult brain play key roles in neurogenesis and learning memory [6, 49]. NK cells have been demonstrated to act in the nervous system, repairing nerve damage, exerting neuroprotective effects, and ameliorating brain damage in Parkinson’s disease [40]. Therefore, in the present study, we investigated whether quercetin exerts neuroprotective effects and enhances cognitive function by modulating immune cells. Quercetin significantly improved cognitive and memory deficits in 10-month-old middle-aged mice. Additionally, we observed an increase in the proportion of NK cells in the peripheral spleen tissue and alterations in the distribution of NK cell subsets, suggesting the promotion of NK cell differentiation. These findings indicate that quercetin can improve cognitive function in aging mice, and that this interaction may be related to the regulation of NK cells. Senescence causes a decrease in the self-renewal capacity of HSCs, an increase in the number of HSCs differentiating from the myeloid lineage, a decrease in the number of HSCs differentiating to the gonadal lineage, and a reduction in the production of immune cells [50]. Here, we found that quercetin increased the proportion of NK cells in peripheral tissues by promoting their self-proliferation and differentiation, rather than by inducing NK cell differentiation from HSCs. Numerous studies have demonstrated that immune cells are capable of traversing the blood-brain barrier to exert immune functions within the central nervous system. For instance, peripheral T cells have been implicated in enhancing cognition and learning in murine models [7, 51], while the presence of NK cells within the hippocampal dentate gyrus has sparked debate among researchers regarding their potential impact on various neurological disorders [44]. Several studies have demonstrated that NK cells possess the ability to facilitate nerve repair following central nervous system injury, mitigate intracerebral damage in Parkinson’s disease, and exert a neuroprotective effect [40]. Moreover, NK cells have shown promise in improving neurodegenerative conditions such as Parkinson’s disease, Lewy body dementia, and Alzheimer’s disease, which are characterized by α-synuclein deposition [9]. Conversely, research has indicated that the senescence of neuroblasts in the aging brain can enhance the cytotoxicity of NK cells, ultimately leading to neurogenesis and cognitive decline [4]. Furthermore, at the Alzheimer’s Association International Conference, NKGen Biotech presented results from its phase 1 clinical trial utilizing NK cell treatment (SNK01) in patients with Alzheimer’s disease. Specifically, 70% of the patients exhibited stable or improved functional scores by the 11th week. Additionally, our current study involving novel object recognition experiments on NK-depleted mice corroborated the cognitive enhancement effects of NK cells, providing further evidence that quercetin’s modulation of NK cells enhances cognitive function in middle-aged mice.

MYH9, which encodes non-muscle myosin heavy chain IIA (NMHC IIA), is a widely-expressed cytoplasmic myosin protein. This class of myosin proteins is ubiquitously present in eukaryotic cells and plays critical roles in various cellular processes that require generation of intracellular mechanical forces and cytoskeletal rearrangements, such as cytokinesis, cell migration, polarization and adhesion, maintenance of cell shape, and signal transduction [52]. The elevated expression of MYH9 protein in immune-related cells indicates a potential significance in immune cell function. MYH9 is known to play a critical role in the modulation of hematopoietic stem cells (HSCs) function and differentiation within the hematopoietic cell lineage. Therefore, conditional knockdown of MYH9 in HSCs leads to a dramatic decrease in the number of HSC cells, severe impairment of hematopoietic function, and reduction of whole blood cells in mice, which ultimately results in reduced survival [38]. Furthermore, MYH9 affects T cell motility and migration [53] and enhances the cytotoxicity of NK cells through binding to solubilized particles after phosphorylation of the protein tail fragment [39]. In our study, the reduction in the proportion of NK cells and in the terminal maturation of NK cells in splenocyte cultures in which the MYH9 protein was downregulated was ameliorated after treatment with quercetin, possibly as a result of its binding to MYH9. However, the specific molecular mechanisms by which MYH9 affects NK cell proliferation and differentiation remain unclear, and therefore warrant further study. However, we speculate that the mechanism of action of quercetin may be related to the role of MYH9 in DNA synthesis [54]. The hippocampus, as the brain region that controls learning and memory, has garnered significant attention in cognition-related research. Hippocampal RNA-seq analysis showed that quercetin mitigates the negative impacts of aging on the brain and improves gene expression patterns in middle-aged mice, making them resemble those in young animals. Thus, the regulation of NK cells by quercetin is associated with its binding to MYH9, which improves cognitive function in aging mice. Furthermore, it is important to acknowledge certain limitations in this study. Specifically, the sample size of mice in the mouse cognition experiment was deemed inadequate, potentially resulting in cognitive results being influenced by individual mouse differences. Despite this limitation, the cognitive tests consistently indicated significant differences between the two groups. Moving forward, larger sample sizes will be utilized in future studies to ensure the acquisition of more dependable statistical data.

In conclusion, our findings suggest that quercetin promotes the proliferation and differentiation of NK cells in peripheral tissues and alleviates cognitive impairment in aging mice by binding to MYH9. However, further investigation is required to elucidate the underlying mechanisms of action.

## Methods

### Animals

C57BL/6 female mice (8-week-old and 10-month-old) were purchased from Jiangsu Biocytogen Co., Ltd (Jiangsu, China). Mice were given 200 µl of 1 mg/kg quercetin (in PBS containing 10% DMSO as solvent) every other day via tail vein injection, and control mice were given the same volume of PBS (containing the same volume of DMSO). The experimental protocols for the animals in this study were approved by the Animal Care Committee of Tongji University and were conducted in accordance with institutional guidelines.

### Open field test (OFT)

A square box (50 × 50 × 30 cm) was used to evaluate anxiety-like behavior. Anxious mice prefer to stay in the corner of the box and make stereotyped movements along the sides. At the beginning of the experiment, mice were placed in the same corner of the box, and their trajectory was tracked and recorded for 15 min. Anxiety was assessed by observing the time spent in the central area.

### Novel object recognition (NOR)

The novel object recognition test evaluates the cognitive and memory abilities of rodents by comparing the times spent exploring familiar and novel (unfamiliar) objects. The test consists of the following three phases: the adaptation phase, the familiarization phase, and the test phase. In the adaptation phase, the mice were free to move in the black square box without any objects for 10 min. In the familiarization phase, two objects (A1 and A2, which have no odor and are fixed and cannot be moved) are placed diagonally at the bottom of the box, approximately 10 cm from the side walls. The mice were placed in the box with their backs facing the objects at an equal distance from the objects and allowed to move freely and explore the objects for 10 min. During the test phase, carried out 1 h after the familiarization phase, object A1 was replaced with a new object, B. The mice were placed again into the box with their backs facing the objects at an equal distance, and their behavior was recorded for 5 min. The times exploring objects A and B were calculated. The preference for the novel objects was assessed using the *cognitive index* (D2), and calculated with the following formula: D2 = N/(N + F), in which N is the exploration time of the novel object, and F is the exploration time of the familiar object.

### Cell preparation, culture, and flow cytometry analysis

The mice were euthanized, and the spleens were removed and rinsed with PBS. The spleens were then ground and filtered through a 70 µM cell filter and lysed using erythrocyte lysate (BD Biosciences).

Spleen mononuclear cells were seeded onto 12-well plates at a density of 1 × 10^6^ per well and treated with different concentrations of quercetin for 24 h (control was an equal volume of DMSO). Flow cytometry was used to quantify the number of T cells (CD3^+^), B cells (CD45R^+^), NK cells (CD3^−^NK1.1^+^) and NK cell subpopulations (CD27^+^CD11b^−^, CD27^+^CD11b^+^, CD27^−^CD11b^+^).

### Isolation and purification of Lin^−^CD117^+^ hematopoietic stem cells

C57BL/6 mice (8 weeks of age) were euthanized by cervical dislocation and sterilized in 75% alcohol for 30 s, preferably allowing the fur to be completely wetted. Next, the femur and tibia were removed and placed in a Petri dish containing pre-chilled PBS for cleaning. The attached muscle tissue was removed as cleanly as possible, and then the cleaned bone was placed in pre-chilled PBS. Next, both ends of the tibia were cut, and the bone marrow was flushed out with a 1-mL syringe until the bone turned white. Bone marrow cells were gently dispersed, and the resulting bone marrow monocyte suspension in PBS was filtered through a 40 μm cell sieve. Subsequently, the bone marrow was centrifuged at 440 × *g* for 5 min, and the supernatant was discarded. Single bone marrow cells were resuspended in 5 mL of red blood cell lysate, and lysis was terminated with 5 mL of pre-cooled PBS after 1–2 min. Next, the cells were centrifuged at 440 × *g* for 5 min, the supernatant was discarded, and 5 mL of pre-cooled PBS was used to wash the cells. An aliquot was taken for counting. After the negative selection of lineage cells using a mouse Lineage Cell Depletion Kit (Miltenyi Biotech), the cells were subjected to positive selection of CD117^+^ cells to obtain the target Lin^−^CD117^+^ HSCs.

### Differentiation culture of NK cells

Lin^−^CD117^+^ hematopoietic stem cells were inoculated at 1 × 10^6^ cells per well into 12-well plates containing RPMI 1640 medium containing 10% fetal bovine serum, 100 U/ml penicillin, 100 ng/ml streptomycin, fIt3L (50 ng/mL), SCF (30 ng/mL) and IL-7 (0.5 ng/mL). After 3 days of culture, the medium was replaced with the same fresh medium. On day 7, the medium was changed to DMEM/F12 medium containing 10% fetal bovine serum, 100 U/ml penicillin, 100 ng/ml streptomycin, IL-15 (30 ng/ml) and IL-2 (5,000 IU/ml), and co-cultured with OP9 stromal cells. Cells were analyzed after 7–11 days of culture, and the medium was replaced with fresh medium every 3 days.

### Identification of proteins that interact with quercetin

Following the extraction of total protein from PBMCs, a bicinchoninic acid assay was used to quantify protein and divide it into six 100-µg aliquots. For each group of three aliquots, methanol was added to the control group, and quercetin was added to the treatment group. After incubating samples at 25 °C for 10 min, Protease K (1:100) was added for 5 min at 25 °C. Subsequently, samples were transferred to a water bath with a temperature greater than 95 °C to completely inactivate Protease K. Next, samples were cooled and placed at room temperature for 5 min. After adding 2% sodium deoxycholate and 200 mM ammonium bicarbonate, DL-dithiothreitol was added and incubated at 37 °C for 30 min. Next, iodoacetamide was added and incubated at room temperature (protected from light) for 45 min. Subsequently, trypsin was added (1:50,

enzyme/protein ratio) and digested overnight in a 37 °C water bath. The following day, 50% trifluoroacetic acid was added to terminate the enzymatic reaction and precipitate the proteins. The enzymatically cleaved peptides were then eluted. After elution, peptides were digested and resuspended in 100 µL 0.1% formic acid to yield a final concentration greater than 2 µg/µL. Finally, mass spectrometry was performed.

### SPR analysis of quercetin and MYH9 protein

The binding ability of quercetin to MYH9 protein was measured by SPR on a BIAcore3000 system (GE Healthcare, Chicago, IL). The MYH9 protein was immobilized on a CM5 chip by its amine group. A 10-µL aliquot of 0.1 mg/mL MYH9 was injected into the flow cell at a rate of 5 µL/min. Successful immobilization of MYH9 was verified by adding approximately 5,000 resonance units to the sensor chip. No MYH9 was injected into the first flow cell as a control. Quercetin was diluted in a buffer containing 20 mM Tris-HCl, 150 mM NaCl and 1 mM TCEP (pH 7.2). After fixation, varying dilutions of quercetin were injected at 30 µL/min for 3 min. After sample injection, the flow buffer was allowed to dissociate by passing over the sensor for 3 min. The sensor surface was regenerated by injecting 20 µL of 10 mM glycine-HCl solution (pH 2.25). Generated data were analyzed at 25 °C using Bioassessment Software 4.1.1 (GE Healthcare).

### Molecular docking

The binding of quercetin and MYH9 protein was simulated with CB-Dock (http://clab.labshare.cn/cb-dock/php/blinddock.php) and AutoDock software. The 3D structure of the MYH9 protein was obtained from UniProt. The CB-Dock calculation program was used to select the highest-scoring results, and then AutoDock was used to predict the cavities. A total of 50 simulated positional predictions were made based on binding affinity ordering. The predicted results with the highest affinity group were put into PyMOL for visualization. Hydrogen bonds formed between quercetin and MYH9 were the proposed binding sites.

### NK cell depletion experiment

C57BL/6 female mice (8 weeks old) were treated with 200 ng of anti-NK1.1 antibody or isotype control IgG2a antibody every 2 days for 6 days, and on the seventh day, the novel object recognition test was conducted.

### RNA-seq analysis

Total RNA from samples was extracted to create an RNA-seq library, which was analyzed by BGI USA (Cambridge, MA) using a BGISEQ-500 sequencer. Raw sequencing reads were cleaned by removing reads containing aptamer or poly-N sequences and reads with low-quality base ratios. Clean reads were stored in FASTQ format and then mapped to the reference genome using the HISAT2 (v2.0.4)/Bowtie2 (v2.2.5) tool. Gene expression levels were calculated using RESM software and normalized to determine gene expression. Heat maps were plotted using GraphPad Prism 8 (GraphPad, San Diego, CA). To gain insight into phenotypic variation, the Kyoto Encyclopedia of Genes and Genomes (KEGG, https://www.kegg.jp/) enrichment analysis of annotated differentially-expressed genes was performed using Phyper (https://en.wikipedia.org/wiki/Hypergeometric_distribution) based on hypergeometric tests. Terms and pathways were corrected for significance levels using Bonferroni’s strict threshold (*Q*-value ≤ 0.05).

### Statistical analysis

Data were analyzed using GraphPad Prism 8.0 and expressed as mean ± standard error of the mean. Statistical comparisons between the two groups were performed using unpaired *t*-test. *P*-values of < 0.05 were considered statistically significant.

### Electronic supplementary material

Below is the link to the electronic supplementary material.

**Supplementary Figure 1**. The effect of quercetin with four different concentrations on NK, T and B cells. (**A**) Representative flow cytometry profiles showing CD3^−^NK1.1^+^ NK cells; Bar graphs for statistical results of splenic NK cells. (**B**) Representative flow cytometry profiles showing CD3^+^ T cells; Bar graphs for statistical results of splenic CD3^+^ T cells. (**C**) Representative flow cytometry profiles showing CD45R^+^ B cells; Bar graphs for statistical results of splenic CD45R^+^ B cells. Data represent mean ± SEM. *P < 0.05. ns, not significant, vs. Ctrl

**Supplementary Figure 2**. The effect of quercetin with three different concentrations on HSCs. Data represent mean ± SEM. *P < 0.05

**Supplementary Figure 3**. Identification of 189 differentially-expressed peptides between the control and quercetin-treated groups. Myh9 peptides appears with high frequency, marked with a red box

## Data Availability

The data that support the findings of this study are available from the corresponding author upon reasonable request. RNA sequencing data are deposited in Gene Expression Omnibus (GEO) with accession number GSE245357.

## Abbreviations

Que: Quercetin
LPS: Lipopolysaccharide
TNF-α: Tumor necrosis factor
HSCs: Hematopoietic stem cells
SPR: Surface plasmon resonance
PCA: Principal component analysis
PPI: Protein–protein interaction
CNS: Central nervous system
GSEA: Gene set enrichment analysis
KEGG: Kyoto Encyclopedia of Genes and Genomes
WGCNA: Weighted gene co-expression network analysis
NMHC IIA: Non-muscle myosin heavy chain IIA
OFT: Open field test
NOR: New object recognition
SEM: Standard error of the mean

## References

[1] Frick KM, Burlingame LA, Arters JA, Berger-Sweeney J (2000). Reference memory, anxiety and estrous cyclicity in C57BL/6NIA mice are affected by age and sex. Neuroscience.

[2] Weng NP (2006). Aging of the immune system: how much can the adaptive immune system adapt?. Immunity.

[3] Yousefzadeh MJ, Flores RR, Zhu Y, Schmiechen ZC, Brooks RW, Trussoni CE, Niedernhofer LJ (2021). An aged immune system drives senescence and ageing of solid organs. Nature.

[4] Garrido A, Cruces J, Ceprián N, Corpas I, Tresguerres JA, De la Fuente M (2019). Social environment improves immune function and redox state in several organs from prematurely aging female mice and increases their lifespan. Biogerontology.

[5] Derecki NC, Cardani AN, Yang CH, Quinnies KM, Crihfield A, Lynch KR, Kipnis J (2010). Regulation of learning and memory by meningeal immunity: a key role for IL-4. J Exp Med.

[6] Radjavi A, Smirnov I, Derecki N, Kipnis J (2014). Dynamics of the meningeal CD4(+) T-cell repertoire are defined by the cervical lymph nodes and facilitate cognitive task performance in mice. Mol Psychiatry.

[7] Brynskikh A, Warren T, Zhu J, Kipnis J (2008). Adaptive immunity affects learning behavior in mice. Brain Behav Immun.

[8] Derecki NC, Quinnies KM, Kipnis J (2011). Alternatively activated myeloid (M2) cells enhance cognitive function in immune compromised mice. Brain Behav Immun.

[9] Earls RH, Menees KB, Chung J, Gutekunst CA, Lee HJ, Hazim MG, Lee JK (2020). NK cells clear α-synuclein and the depletion of NK cells exacerbates synuclein pathology in a mouse model of α-synucleinopathy. Proc Natl Acad Sci U S A.

[10] Ross JA, Kasum CM (2002). Dietary flavonoids: bioavailability, metabolic effects, and safety. Annu Rev Nutr.

[11] Manca ML, Castangia I, Caddeo C, Pando D, Escribano E, Valenti D, Manconi M (2014). Improvement of quercetin protective effect against oxidative stress skin damages by incorporation in nanovesicles. Colloids Surf B Biointerfaces.

[12] Abel AM, Yang C, Thakar MS, Malarkannan S (2018). Natural killer cells: Development, Maturation, and clinical utilization. Front Immunol.

[13] Chou CC, Yang JS, Lu HF, Ip SW, Lo C, Wu CC, Chen DR (2010). Quercetin-mediated cell cycle arrest and apoptosis involving activation of a caspase cascade through the mitochondrial pathway in human breast cancer MCF-7 cells. Arch Pharm Res.

[14] Lee TJ, Kim OH, Kim YH, Lim JH, Kim S, Park JW, Kwon TK (2006). Quercetin arrests G2/M phase and induces caspase-dependent cell death in U937 cells. Cancer Lett.

[15] Zhou J, Fang L, Liao J, Li L, Yao W, Xiong Z, Zhou X (2017). Investigation of the anti-cancer effect of quercetin on HepG2 cells in vivo. PLoS ONE.

[16] Ishisaka A, Ichikawa S, Sakakibara H, Piskula MK, Nakamura T, Kato Y, Terao J (2011). Accumulation of orally administered quercetin in brain tissue and its antioxidative effects in rats. Free Radic Biol Med.

[17] Zaplatic E, Bule M, Shah SZA, Uddin MS, Niaz K (2019). Molecular mechanisms underlying protective role of quercetin in attenuating Alzheimer’s disease. Life Sci.

[18] Geng L, Liu Z, Zhang W, Li W, Wu Z, Wang W, Liu GH (2019). Chemical screen identifies a geroprotective role of quercetin in premature aging. Protein Cell.

[19] Xu M, Pirtskhalava T, Farr JN, Weigand BM, Palmer AK, Weivoda MM, Kirkland JL (2018). Senolytics improve physical function and increase lifespan in old age. Nat Med.

[20] Chang HC, Yang YR, Wang PS, Wang RY (2014). Quercetin enhances exercise-mediated neuroprotective effects in brain ischemic rats. Med Sci Sports Exerc.

[21] Ishikawa Y, Kitamura M (2000). Anti-apoptotic effect of quercetin: intervention in the JNK- and ERK-mediated apoptotic pathways. Kidney Int.

[22] Dong F, Wang S, Wang Y, Yang X, Jiang J, Wu D, Yao R (2017). Quercetin ameliorates learning and memory via the Nrf2-ARE signaling pathway in d-galactose-induced neurotoxicity in mice. Biochem Biophys Res Commun.

[23] Tanigawa S, Fujii M, Hou DX (2007). Action of Nrf2 and Keap1 in ARE-mediated NQO1 expression by quercetin. Free Radic Biol Med.

[24] Boesch-Saadatmandi C, Pospissil RT, Graeser AC, Canali R, Boomgaarden I, Doering F, Rimbach G (2009). Effect of quercetin on paraoxonase 2 levels in RAW264.7 macrophages and in human monocytes–role of quercetin metabolism. Int J Mol Sci.

[25] Sun GY, Chen Z, Jasmer KJ, Chuang DY, Gu Z, Hannink M, Simonyi A (2015). Quercetin attenuates inflammatory responses in BV-2 microglial cells: role of MAPKs on the Nrf2 pathway and induction of Heme Oxygenase-1. PLoS ONE.

[26] Sriraksa N, Wattanathorn J, Muchimapura S, Tiamkao S, Brown K, Chaisiwamongkol K (2012). Cognitive-enhancing effect of quercetin in a rat model of Parkinson’s disease induced by 6-hydroxydopamine. Evid Based Complement Alternat Med.

[27] Khan H, Singh A, Thapa K, Garg N, Grewal AK, Singh TG (2021). Therapeutic modulation of the phosphatidylinositol 3-kinases (PI3K) pathway in cerebral ischemic injury. Brain Res.

[28] Wang S, Su R, Nie S, Sun M, Zhang J, Wu D, Moustaid-Moussa N (2014). Application of nanotechnology in improving bioavailability and bioactivity of diet-derived phytochemicals. J Nutr Biochem.

[29] Sun SW, Yu HQ, Zhang H, Zheng YL, Wang JJ, Luo L (2007). Quercetin attenuates spontaneous behavior and spatial memory impairment in d-galactose–treated mice by increasing brain antioxidant capacity. Nutr Res.

[30] Manjeet KR, Ghosh B (1999). Quercetin inhibits LPS-induced nitric oxide and tumor necrosis factor-alpha production in murine macrophages. Int J Immunopharmacol.

[31] Bureau G, Longpré F, Martinoli MG (2008). Resveratrol and quercetin, two natural polyphenols, reduce apoptotic neuronal cell death induced by neuroinflammation. J Neurosci Res.

[32] Yu CS, Lai KC, Yang JS, Chiang JH, Lu CC, Wu CL, Chung JG (2010). Quercetin inhibited murine leukemia WEHI-3 cells in vivo and promoted immune response. Phytother Res.

[33] Gong X, Shen H, Guo L, Huang C, Su T, Wang H, Liu H (2023). Glycyrrhizic acid inhibits myeloid differentiation of hematopoietic stem cells by binding S100 calcium binding protein A8 to improve cognition in aged mice. Immun Ageing.

[34] Wu Y, Zhu J, Liu H, Liu H (2021). Licochalcone A improves the cognitive ability of mice by regulating T- and B-cell proliferation. Aging.

[35] Lee J, Zhang T, Hwang I, Kim A, Nitschke L, Kim M, Kim S (2015). Epigenetic modification and antibody-dependent expansion of memory-like NK cells in human cytomegalovirus-infected individuals. Immunity.

[36] Erick TK, Brossay L (2016). Phenotype and functions of conventional and non-conventional NK cells. Curr Opin Immunol.

[37] Pang WW, Schrier SL, Weissman IL (2017). Age-associated changes in human hematopoietic stem cells. Semin Hematol.

[38] An Q, Dong Y, Cao Y, Pan X, Xue Y, Zhou Y, Ma F. Myh9 plays an essential role in the survival and maintenance of hematopoietic Stem/Progenitor cells. Cells. 2022;11(12). 10.3390/cells11121865 10.3390/cells11121865 PMC9221478 35740994

[39] Sanborn KB, Mace EM, Rak GD, Difeo A, Martignetti JA, Pecci A, Orange JS (2011). Phosphorylation of the myosin IIA tailpiece regulates single myosin IIA molecule association with lytic granules to promote NK-cell cytotoxicity. Blood.

[40] Earls RH, Lee JK (2020). The role of natural killer cells in Parkinson’s disease. Exp Mol Med.

[41] Burrack KS, Huggins MA, Taras E, Dougherty P, Henzler CM, Yang R, Alter S, Jeng EK, Wong HC, Felices M, Cichocki F, Miller JS, Hart GT, Johnson AJ, Jameson SC, Hamilton SE (2018). Interleukin-15 Complex Treatment protects mice from cerebral malaria by inducing Interleukin-10-Producing Natural Killer cells. Immunity.

[42] Ghasemi R, Lazear E, Wang X, Arefanian S, Zheleznyak A, Carreno BM, Higashikubo R, Gelman AE, Kreisel D, Fremont DH, Krupnick AS (2016). Selective targeting of IL-2 to NKG2D bearing cells for improved immunotherapy. Nat Commun.

[43] Hölscher C (2003). Time, space and hippocampal functions. Rev Neurosci.

[44] Jin W-N, Shi K, He W, Sun J-H, Van Kaer L, Shi F-D, Liu Q (2021). Neuroblast senescence in the aged brain augments natural killer cell cytotoxicity leading to impaired neurogenesis and cognition. Nat Neurosci.

[45] Karimipour M, Rahbarghazi R, Tayefi H, Shimia M, Ghanadian M, Mahmoudi J, Bagheri HS (2019). Quercetin promotes learning and memory performance concomitantly with neural stem/progenitor cell proliferation and neurogenesis in the adult rat dentate gyrus. Int J Dev Neurosci.

[46] Li H, Chen FJ, Yang WL, Qiao HZ, Zhang SJ (2021). Quercetin improves cognitive disorder in aging mice by inhibiting NLRP3 inflammasome activation. Food Funct.

[47] Xu M, Huang H, Mo X, Zhu Y, Chen X, Li X, Liu L (2021). Quercetin-3-O-Glucuronide alleviates cognitive deficit and toxicity in Aβ(1–42) -Induced AD-Like mice and SH-SY5Y cells. Mol Nutr Food Res.

[48] Ogrodnik M, Evans SA, Fielder E, Victorelli S, Kruger P, Salmonowicz H, Jurk D (2021). Whole-body senescent cell clearance alleviates age-related brain inflammation and cognitive impairment in mice. Aging Cell.

[49] Ziv Y, Ron N, Butovsky O, Landa G, Sudai E, Greenberg N, Schwartz M (2006). Immune cells contribute to the maintenance of neurogenesis and spatial learning abilities in adulthood. Nat Neurosci.

[50] Dykstra B, Olthof S, Schreuder J, Ritsema M, de Haan G (2011). Clonal analysis reveals multiple functional defects of aged murine hematopoietic stem cells. J Exp Med.

[51] Kipnis J, Cohen H, Cardon M, Ziv Y, Schwartz M (2004). T cell deficiency leads to cognitive dysfunction: implications for therapeutic vaccination for schizophrenia and other psychiatric conditions. Proc Natl Acad Sci U S A.

[52] Pecci A, Ma X, Savoia A, Adelstein RS (2018). MYH9: structure, functions and role of non-muscle myosin IIA in human disease. Gene.

[53] Jacobelli J, Friedman RS, Conti MA, Lennon-Dumenil AM, Piel M, Sorensen CM, Krummel MF (2010). Confinement-optimized three-dimensional T cell amoeboid motility is modulated via myosin IIA-regulated adhesions. Nat Immunol.

[54] Nangia-Makker P, Shekhar MPV, Hogan V, Balan V, Raz A (2022). MYH9 binds to dNTPs via deoxyribose moiety and plays an important role in DNA synthesis. Oncotarget.

