# Supplementary figures and images for: Quercetin promotes the proportion and maturation of NK cells by binding to MYH9 and improves cognitive functions in aged mice
Source: Immun Ageing. 2024 May 10;21:29. doi: 10.1186/s12979-024-00436-1 (PMC11084035; doi:10.1186/s12979-024-00436-1)

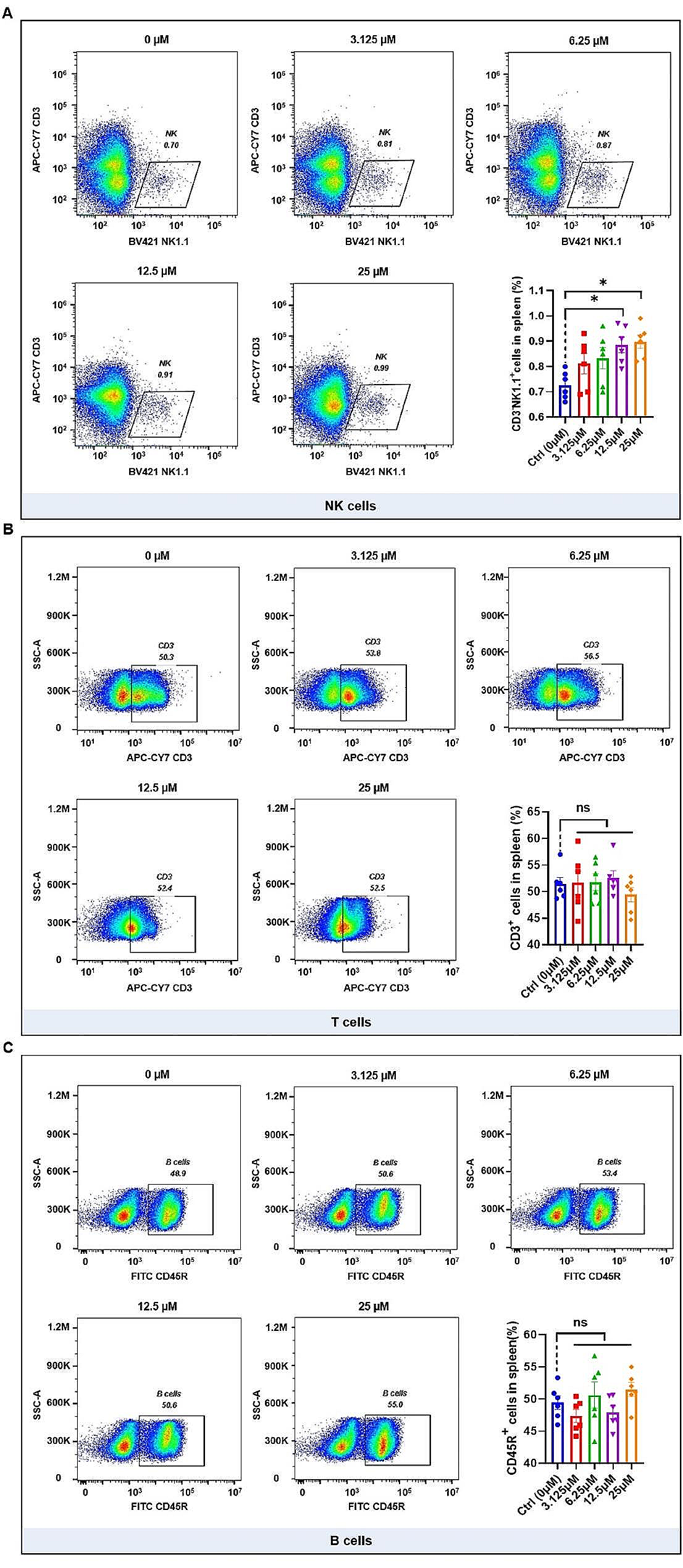

Supplement: Supplementary file 1 — Supplementary Figure 1. The effect of quercetin with four different concentrations on NK, T and B cells. (A) Representative flow cytometry profiles showing CD3−NK1.1+ NK cells; Bar graphs for statistical results of splenic NK cells. (B) Representative flow cytometry profiles showing CD3+ T cells; Bar graphs for statistical results of splenic CD3+ T cells. (C) Representative flow cytometry profiles showing CD45R+ B cells; Bar graphs for statistical results of splenic CD45R+ B cells. Data represent mean ± SEM. *P < 0.05. ns, not significant, vs. Ctrl [file 12979_2024_436_MOESM1_ESM.png]

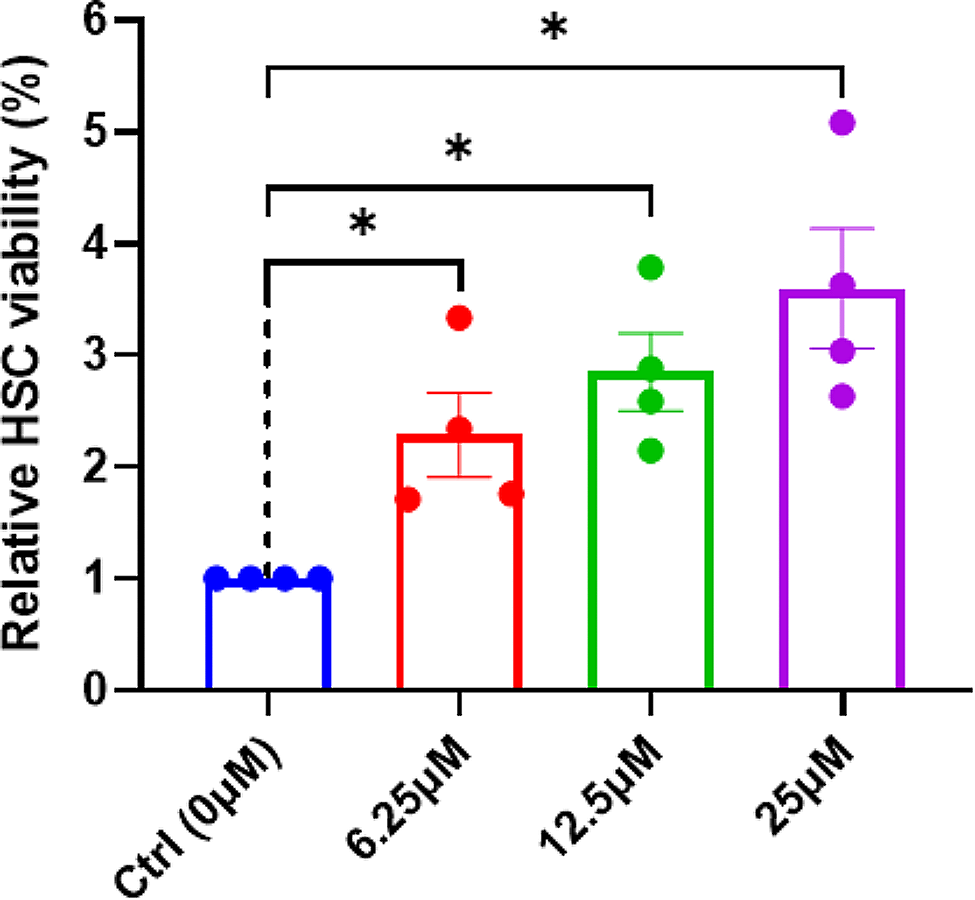

Supplement: Supplementary file 2 — Supplementary Figure 2. The effect of quercetin with three different concentrations on HSCs. Data represent mean ± SEM. *P < 0.05 [file 12979_2024_436_MOESM2_ESM.png]

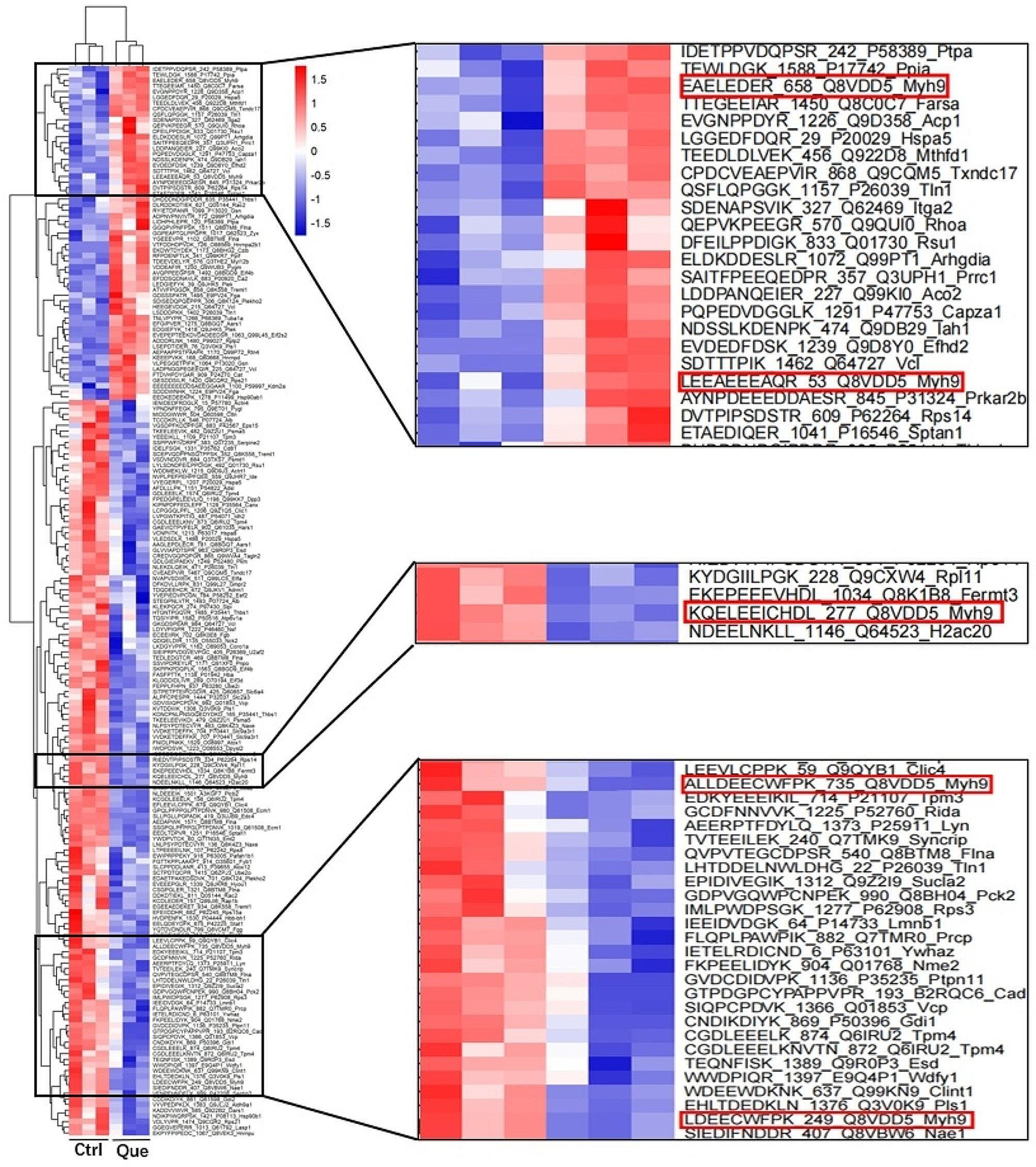

Supplement: Supplementary file 3 — Supplementary Figure 3. Identification of 189 differentially-expressed peptides between the control and quercetin-treated groups. Myh9 peptides appears with high frequency, marked with a red box [file 12979_2024_436_MOESM3_ESM.png]
